# Simultaneous Pre-Concentration and HPLC-MS/MS Quantification of Phycotoxins and Cyanotoxins in Inland and Coastal Waters

**DOI:** 10.3390/ijerph17134782

**Published:** 2020-07-03

**Authors:** Francesca Merlo, Federica Maraschi, Davide Piparo, Antonella Profumo, Andrea Speltini

**Affiliations:** 1 Department of Chemistry, University of Pavia, 27100 Pavia, Italy; francesca.merlo02@universitadipavia.it (F.M); federica.maraschi@unipv.it (F.M); davide.piparo01@universitadipavia.it (D.P.); 2 Department of Drug Sciences, University of Pavia, 27100 Pavia, Italy; andrea.speltini@unipv.it

**Keywords:** single-cartridge SPE, Strata™-X, cyanobacterial and algal toxins, multi-class method, environmental waters

## Abstract

The purpose of this study was to set up a sensitive method for the simultaneous determination of phycotoxins and cyanotoxins—Emerging pollutants with different structures and harmful properties (hepatotoxicity, neurotoxicity and cytotoxicity)—In environmental waters. Due to the low concentrations detected in these samples, a pre-concentration step is required and here it was performed in a single step with a commercial cartridge (Strata™-X), achieving enrichment factors up to 200 and satisfactory recovery (R = 70–118%) in different aqueous matrices. After solid-phase extraction (SPE), toxins were separated and quantified by High Performance Liquid Chromatography- Heated ElectroSpray Ionisation Tandem Mass Spectrometry (HPLC-HESI-MS/MS) in Multiple Reaction Monitoring (MRM) mode. An analytical evaluation of the proposed method was done based on the analytical figures of merit, such as precision and trueness, linearity, selectivity, and sensitivity, and it turned out to be a robust tool for the quantification of ng L^−1^ levels, phycotoxins and cyanotoxins in both freshwater and saltwater samples.

## 1. Introduction

**Figure 1:**
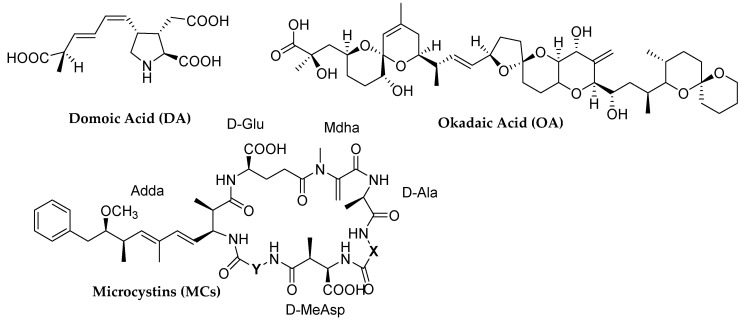
Molecular structures of the target phycotoxins and cyanotoxins.

Global warming and anthropogenically-caused eutrophication of aquatic environments are linked with increased frequency and magnitude of harmful algal blooms [1,2]. This phenomenon has been reported worldwide and it is generally accompanied with the release of natural toxins, such as phycotoxins and cyanotoxins. The production of these harmful compounds may compromise water quality and its consequent use for drinking and recreational purposes. Moreover, the presence of these biotoxins is often associated with mass mortality and morbidity event occurrences in aquatic animals [2]. In this work, we focalized our attention on two phycotoxins of different hydrophilicity (Domoic acid, DA, and Okadaic acid, OA) and four Microcystins (MCs) belonging to the family of cyanotoxins, biotoxins all found worldwide in inland and coastal water environments (Figure 1).

DA is an algal neurotoxin produced by different species of marine diatoms of the genus *Pseudo-nitzshia*, whose blooms typically occur in periods of low temperature and in the presence of low light, unlike most species of algae. As shown in Figure 1, DA is a bicarboxylic amino acid, which can exist in five forms depending on the pH of the solution (pKa = 1.85-4.47-4.75-10.60). Figure S1 (Supplementary Material) reports the structure of DA (major congener) and its nine possible isomers [3,4]. It is structurally analogous to two neurotransmitter amino acids, L-glutamic acid and kainic acid; therefore, DA is able to interfere with neurotransmission mechanisms by irreversible binding to glutamate receptors. Moreover, DA is responsible for amnesic shellfish poisoning (ASP), a pathology observed in humans after the consumption of contaminated bivalves. For this reason, the acceptable limit value to protect human health is 20 μg of DA/g of wet tissue [5,6,7,8].

OA is a marine toxin belonging to a group of polyketides and is produced by dinoflagellates of the genera *Prorocentrum* and *Dinophysis*, which are widespread worldwide especially in temperate and tropical waters. Regarding its structure (see Figure 1), OA is a cyclic polyether C38 fatty acid with a remarkable variety of functionalities and structural features. OA contains 17 stereogenic centers, 3 spiroketal moieties, a trans alkene, a 1-disubstituted exocyclic alkene, an α- methyl, α-hydroxy carboxylic acid, and 3 secondary alcohols [9]. The toxicity of OA is related to the inhibition of phosphatase proteins PP1 and PP2A, selective for serine and threonine. High amounts of OA can accumulate in bivalve mollusks, causing diarrheic shellfish poisoning (DSP) in humans [10,11,12].

MCs are hepatic cyanotoxins, produced by prokaryotic and photosynthetic bacteria called cyanobacteria. Although they are widespread mainly in freshwater, they have also recently even been detected in marine ecosystems [13,14]. Their structure consists of a cyclic heptapeptide which contains D-alanine, D-eritro-methyl aspartate, D-glutamate, N-methyldehydro-alanine, a characteristic aromatic amino acid known as Adda, and two variable amino acids (X and Z) in positions 2 and 4 (Figure 1). Their names and their characteristics depend on these two amino acids. In this work, four MC variants were considered and listed in the Table S1 (Supplementary Material). Toxicologically, the amino acid Adda made MCs potent inhibitors of PP1 and PP2A, proteins of vital importance in cellular control and intracellular structure.

The World Health Organization (WHO) established a guideline of 1 µg L^−1^ in drinking water for total MC-LR, the most frequent MC variant.

Due to their toxicological activities on the environment, animals and humans, the assessment of cyano- and phyco-toxins is important in both fresh and marine waters, thus it is increasingly necessary to have sensitive analytical methods for their determination at low concentration levels (low ng L^−1^ to μg L^−1^ levels). However, the determination of toxins, and in particular of MCs, is challenging because of lack of analytical standards, matrix interferences and their different chemical properties (such as polarity). In this context, high-performance liquid chromatography tandem mass spectrometry (HPLC-MS/MS) is undoubtedly a powerful tool since it can provide detection/differentiation/quantitation of MC congeners; however, a sample pre-treatment is often required to pre-concentrate these compounds to obtain limits of detection (LODs) in the ng L^−1^ range. Among the common techniques, solid phase extraction (SPE) and magnetic solid phase extraction (MSPE) are often used due to their attractive properties such as flexibility, high enrichment factor (EF), low consumption of reagents, and the possible choice among different sorbent materials. However, most of the existing literature data propose analytical methods for single class determination of toxins in water [15,16,17,18,19,20,21,22,23], whereas only few methods focus on simultaneous determination of the various classes of cyanotoxins and phycotoxins. Moreover, the use of a single SPE is not enough to provide quantitative recoveries for all the classes, so the multi-cartridge SPE approach was applied [24,25]. Therefore, the choice of the sorbent used for toxin enrichment plays a key role in extraction efficiency and pre-concentration.

The purpose of this study was indeed to develop an easy and sensitive analytical multi-toxin method based on SPE followed by HPLC-HESI-MS/MS for simultaneous pre-concentration and determination of total phycotoxins and cyanotoxins in fresh and salt waters. To achieve this goal, the commercial cartridge Strata™-X—A vinylpyrrolidone chemically modified divinylbenzene polymeric sorbent which offers strong retention of neutral, acidic, or basic compounds—Was successfully employed for the first time for the simultaneous extraction of hydrophilic and lipophilic biotoxins from environmental waters with different salinity and complexity (lake, river and sea water samples).

## 2. Materials and Methods

### 2.1. Chemicals and Materials

All chemicals were reagent grade or higher in quality. Formic acid (FA) and ammonium hydroxide solution (NH_4_OH) were purchased from Sigma-Aldrich (Milan, Italy). HPLC gradient grade methanol (MeOH), acetonitrile (ACN) and ultrapure water (H_2_O) were supplied by Carlo Erba (Cornaredo, Italy). Strata™-X cartridges (200 mg, 3 mL) were purchased from Phenomenex (Castel Maggiore, Italy). DA (≥90%), OA sodium salt (MQ 100) and analytical grade standard MC-RR, MC-LR, MC-YR and MC-LW solutions were supplied by Sigma-Aldrich (Milan, Italy). Individual stock standard solutions of all analytes (1 μg mL^−1^) were prepared in MeOH, stored in the dark (−20 °C) and renewed weekly.

### 2.2. Water Samples

Tap water was from the Pavia municipal waterworks. Environmental samples were collected in northern Italy from the Mergozzo and Garda Lakes, the Staffora River and the Ligurian Sea, and the physical-chemical parameters were measured for each sample, as reported in Table S2, (Supplementary Material). In order to facilitate cellular lysis and thus to determine the total content of each toxin, the samples were frozen (−20 °C), then thawed and sonicated for at least one cycle [26,27].

### 2.3. SPE Procedure

The Strata™-X cartridge was conditioned with 5 mL of MeOH, 5 mL of deionized H_2_O and lastly with 3 mL of H_2_O + 0.1% *v*/*v* FA. The water samples (500 mL), previously acidified with FA to achieve pH~3, were loaded onto the SPE cartridge at a flow rate of 5 mL min^−1^ by means of a vacuum manifold (Resprep manifold, Restek Corporation, Bellefonte, USA). After drying under a vacuum for 5 min, the analytes were eluted (1 mL min^−1^) in a single fraction with 2.5 mL MeOH + 1% *v*/*v* FA.

### 2.4. HPLC-HESI-MS/MS Analysis

HPLC apparatus Agilent 1260 Infinity, coupled with an Agilent 6460C MS spectrometer HESI-MS/MS system (Cernusco sul Naviglio, Italy), was used for quantitative analysis. For chromatographic separation, the Zorbax Eclipse Plus C18 (2.1 × 150 mm, 3.5 μm) column, preceded by a pre-column C18 (Supelco Sigma-Aldrich), both thermostated at 30 °C (± 0.8 °C), were used. Elution was performed by (A) H_2_O and (B) ACN both containing 0.5% FA, added to improve the ionization of the compounds [24]. The elution program started at 95% A (maintained for 3 min), decreasing to 80% A in 1 min (maintained for 2 min), to 65% A in 1 min (maintained for 7 min), to 30% A in 14 min and to 10% in 1 min, and then to 2% A in 1 min (maintained for 10 min). The initial conditions were re-established by a 10-min equilibration time. The flow rate was 0.2 mL min^−1^ and the sample injection volume was 10 μL.

The MS spectrometer was tuned up by direct injection of individual analyte solutions (0.5 mg L^−1^ in MeOH) and ionization of the analytes was carried out in positive mode. The operating parameters of the MS detector were: drying gas (N_2_) and sheath gas temperature 350 °C and 355 °C, respectively; drying gas and sheath gas flow 12 L min^−1^; nebulizer 50 psi; capillary voltage 5000 V positive, 0 V negative; nozzle voltage 1000 V positive, 0 V negative; electron multiplier voltage (EMV) 0 V both positive and negative; cell accelerated voltage 4 V positive, 1 V negative. The Multiple Reaction Mode (MRM) mode was adopted for the quantification of the target toxins, using the highest and characteristic precursor/product ion transitions of each compound obtained from the MS/MS spectra. A typical MRM chromatogram of a standard solution (200 μg L^−1^) is shown in Figure S2 (Supplementary Material).

### 2.5. Analytical Evaluation of the SPE Procedure Followed by HPLC-HESI-MS/MS

The entire analytical procedure was validated based on the main figures of merit, viz. trueness, precision, selectivity, linearity, detection, and quantification limits.

No certified reference materials were available, thus trueness and precision of the method were studied through the recovery of each toxin in tap, lake, river and sea waters, working on fortified samples in the concentration range 100–400 ng L^−1^ (*n* = 3). Within-laboratory precision was calculated through relative standard deviation (RSD%). Calibration curves were constructed from eight standard solutions, prepared in a solvent (MeOH + 1% *v*/*v* FA), in the dynamic range from 2.5 μg L^−1^ to 250 μg L^−1^. Each concentration level was analyzed in duplicate. A Mandel’s Test was performed to check which regression model better fit the data in this range. A “T-value” was calculated according to the Equation (1):

(1) $$TV=\frac{\left(N-2\right)\cdot S_{y}/x_{1}^{2}-\left(N-3\right)\cdot S_{y}/x_{2}^{2}}{S_{y}/x_{2}^{2}}$$

where *N* is the number of calibration levels, and $s_{y/x_{1}}$ and $s_{y/x_{2}}$ are the standard errors of the straight-line and the quadratic regression mode. The obtained “T-values” were compared with the critical value F_1-α, 1, N-3_ tabulated (one-tailed test), relative to the confidence level considered (95%).

Alteration of ionization efficiency (ion suppression or improvement) of the target analyte in the presence of co-eluting sample constituents, co-extracted during SPE, could cause some matrix effects (ME), with consequent errors and inaccurate results. In this work, to overcome these problems, the matrix-matched calibration curves were constructed, preparing the standards in the eluate obtained from pre-concentration of the blank sample (500 mL of lake, river and sea waters), and used for calculating ME and Method Detection and Quantification Limits (MDLs and MQLs, respectively). The first one was evaluated by comparing the slopes of those matrix calibration curves (b_m_) against those observed in neat solvent (b_s_), according to the following Equation (2):

(2) $$\mathrm{ME}=100\times b_{m}/b_{s}$$

For each analyte, MDL and MQL were calculated as three and ten times, respectively, the ratio of the baseline noise away from the peak tails of the lowest-concentration standard, and the regression line slope (b_m_), taking into consideration the EF [28,29,30].

## 3. Results and Discussion

### 3.1. SPE Procedure

The commercial cartridge Strata™-X is a functionalized polymeric sorbent that contains N-vinylpyrrolidone and it is widely used as a mixed-mode solid-phase, able to strongly retain neutral, acidic, or basic organic compounds via different, combined retention mechanisms, such as pi–pi bonding, hydrogen bonding (dipole–dipole interactions), and hydrophobic interactions. Based on these properties, Strata™-X was here tested for the multi-class extraction of phycotoxins and cyanotoxins from water.

**Figure 2:**
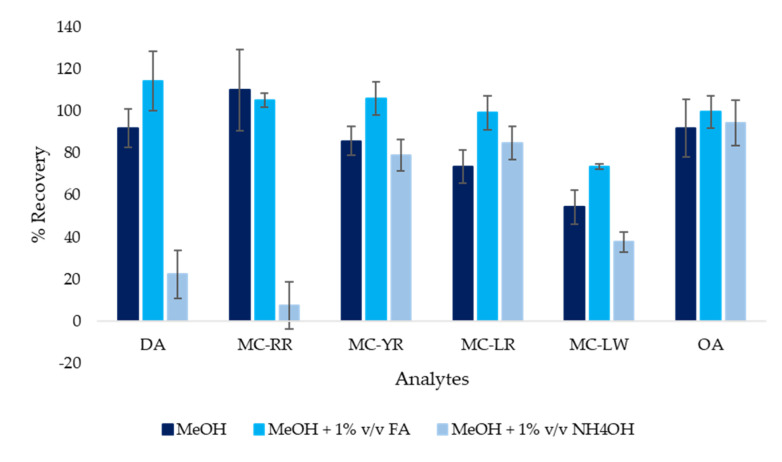
Comparison of the solvents tested for analyte elution (*n* = 3).

Preliminary pre-concentration tests were undertaken on tap water samples (250 mL) enriched with 800 ng L^−1^ of each analyte to evaluate the role of different parameters, namely extraction pH and composition of the cartridge eluting phase. Analyte adsorption was calculated as the difference between the quantity loaded and that determined in the percolate, after its evaporation in a vacuum. To this aim, the percolate residue was taken up first with 2 mL of tridistilled water to dissolve any traces of DA, the most hydrophilic compound, and then with 2 mL of MeOH to best dissolve the fractions of MCs and OA eventually not retained on the solid phase. Adsorption resulted as pH independent for all analytes except for DA, which needed acidic conditions to be retained on the cartridge, as just previously reported [25]. Therefore, extraction needed to be carried out at a pH close to 3, which was achieved using FA. The elution step was then studied testing MeOH as the eluent, with good recoveries for most of the analytes, except for MC-LW, which was not quantitatively desorbed (R < 70%), even when the eluent volume was increased from 2.5 mL to 4 mL (data not shown). To improve recovery, methanolic elution solvents with FA (1% *v*/*v*) or NH_4_OH (1% *v*/*v*) were then tested. Based on the results shown in Figure 2, MeOH + 1% *v*/*v* NH_4_OH failed to desorb the majority of analytes, so it was excluded as an eluent. Higher recoveries were observed for MC-LW using MeOH + 1% *v*/*v* FA as the eluent (R > 75%), in agreement with Zervou et al. [24]. Moreover, the use of MeOH + 1% *v*/*v* FA gave an improvement in the chromatographic performance, so it was selected as the eluting solvent (2.5 mL).

In view of these findings, subsequent trials were performed (*n = 3*) by pre-concentrating a larger volume (500 mL) of tap water enriched at two different concentration levels (100 ng L^−1^ and 400 ng L^−1^). All results, gathered in Table S3, show unchanged performances (70% < R < 120%), RSD < 10% and EF equal to 200. These data point out the applicability of the proposed method for quantification of the total concentration of biotoxins in drinking waters at levels lower than the limit proposed by the WHO for MC-LR (1 μg L^−1^).

As previously reported, the assessment of phycotoxins and cyanotoxins in both freshwater and saltwater is increasingly important, so the final analytical procedure (500 mL, pH~ 3, 2.5 mL MeOH + 1% *v*/*v* FA) was applied for enrichment in environmental matrices (Mergozzo Lake water, Staffora River water and seawater from the Ligurian Sea). Despite the high salt content of seawater and in general the complexity of the investigated matrices, the SPE procedure was performed without any problems of cartridges clogging or requiring an additional clean-up. The absence of the analytes was verified by recovery tests and quantification by the standard addition method, and all the analytes were below the MDLs. As shown in Table 1, good performance was obtained by pre-concentrating each sample enriched at different concentrations (100 ng L^−1^ and 400 ng L^−1^), in a single step with no need for clean-up, with recoveries (*n* = 3) in the range 82–118% for lake water, 70–110% for river water and 70–101% seawater.

These data highlighted that the proposed method allows a pre-concentration of toxins with different chemical properties (polarity, hydrophilicity and molecular weight) from water samples with high differences in chemical–physical properties, with a significant reduction in time and cost of analysis compared to the methods recently proposed in the literature [24,25]. Indeed, the analytical protocol here shown entails the use of a single cartridge, avoiding the dual sequential SPE cartridge assembly, the use of a large volume of halogenated solvents [24,25], and the clean-up generally necessary with samples with a high salt content.

### 3.2. HPLC-HESI-MS/MS Analysis

The Zorbax Eclipse Plus C18 column (150 × 2.1 mm, 3.5 μm) proved to be suitable for simultaneous separation of all analytes, which were baseline-separated with symmetrical and narrow peaks, also at a high percentage of water (95% A at the beginning of the gradient), as shown in Figure S2 (Supplementary Material). The retention times of each compound are reported in Table 2. The MS spectrometer was tuned up by direct injection of individual toxin solutions (0.5 mg L^−1^ in MeOH) and ionization of compounds was carried out in positive mode. Based on the literature and on the experimental results, the possible structures of each ion are reported in Table 2.

As reported before by Zervou et al. [24], the single protonated molecular ion [M+H]^+^ is observed as a precursor ion for all analytes, except for MC-RR. DA and OA have different oxygenated groups (-COOH and -OH) on which protonation can easily occur. With regard to MC-LR and MC-YR, the formation of this ion is due to the presence of an arginine residue, which is the favoured site for protonation. MC-RR instead presents as a precursor—The doubly protonated ion [M + 2H]^2+^, because it contains two arginine residues in its molecular structure. Since MC-LW does not contain the arginine residue, it can be hypothesized that the protonation site for the precursor ion is the methoxy group of the residue Adda [24].

### 3.3. Analytical Evaluation of the SPE Procedure Followed by HPLC-HESI-MS/MS

As reported in Section 2.5, precision and trueness were investigated by recovery tests at three different concentration levels using blank samples (analytes concentration lower than MDLs) from lake, river and sea waters. Recoveries, expressed as percentages, were calculated as the ratio of the measured concentration and the theoretical one, considering EF. As reported in Table 1, satisfactory performances were obtained in the different water matrices, with recovery between 70% and 118%.

Intra- and inter-day precision showed RSDs below 16% in lake water, 15% in river water and 10% in sea water.

The evaluation of the (non) linearity of the regression model was carried out by the Mandel’s Fitting test. This highlighted that the Ordinary Linear Least Squares Regression (OLLSR) could be applied for all the compounds except for OA. For this analyte, a quadratic model better fits the data in the range 2.5–250 μg L^−1^, while in the range 2.5–80 μg L^−1^ OLLSR could be used.

As detailed in Section 2.5, the presence of ME was assessed by comparing the slopes of matrix-matched calibration curves and those of the calibration lines obtained in pure solvent. The complexity and the salinity of the seawater resulted in a considerableME, as reported in Table S4. ME is present also in lake and river water, even if more moderately. Based on these findings, the matrix-matched calibration method was adopted for the quantification of the analytes and for the calculation of MDLs and MQLs. As reported in Table 3, MDLs and MQLs are in the ppt levels, up to thirty times below the WHO trigger value.

Selectivity is guaranteed by MRM detection, which allows identification/quantification of the target toxins by using the two most intense transitions, and by the absence of interfering peaks close to the retention time of the target analytes in pre-concentrated blank matrices. The results in terms of recovery, selectivity and sensitivity, highlight that the proposed method is suitable for environmental monitoring and for controlling drinking water contamination. Therefore, the analytical protocol was applied to the analysis of a sample collected in October 2019 in the Garda Lake, one of the most important Italian lakes for recreational purposes. In this sample, the target analytes were not detected, but it has been well documented that the presence of toxins highly depends on the sampling seasonality and on a complex suite of interacting environmental factors [27,31]. The proposed method could be applied for long-term monitoring activity, providing a multi-toxin profile in freshwater and saltwater, helpful for the appraisal of potential risks to the environment and human health.

## 4. Conclusions

Simultaneous extraction of cyanotoxins and phycotoxins from environmental waters with different salinity and complexitywas achieved by a single-cartridge SPE (Strata™-X), for the first time employed for multi-class enrichment of these biotoxins, followed by their one run determination by HPLC-HESI–MS/MS i(MRM mode), saving time and reagents. The method was comprehensively and successfully evaluated in terms of trueness, precision, linearity, and sensitivity. The obtained results proved that it could be a suitable alternative to previous methods for the accurate and simultaneous quantification of multi-class toxins at ng L^−1^ concentration levels in tap, river, lake and sea waters.

## Figures and Tables

**Table 1:** Mean recovery (%) in the water samples (*n* = 3).

	Mean Recoveries (%)					
Toxin	Lake Water		River Water		Sea Water	
	400 ng L^−1^	100 ng L^−1^	400 ng L^−1^	100 ng L^−1^	400 ng L^−1^	100 ng L^−1^
DA	82	92	70	86	79	87
MC-RR	89	90	71	95	84	101
MC-YR	106	114	110	89	97	87
MC-LR	112	118	96	87	96	95
MC-LW	104	108	71	71	70	86
OA	97	113	82	103	94	93

For lake water: RSD < 16%; for river water: RSD < 15%; for seawater: RSD < 10%.

**Table 2:** Instrumental conditions.

Toxin	Retention Time (min)	*m/z* Precursor Ion	*m/z* and Hypothesized Formula for Product Ion*	Dwell Time (ms)	Fragmentor Voltage (V)	Collision Energy (V)
DA	16.4	312.1 [M+H]^+^	266.5 [M+H-HCOOH]^+^	100	50	15
			161.2 [(COOH)_2_C_4_H_7_NH_2_]^+^	100	50	20
MC-RR	20.1	519.9 [M+2H]^2+^	135.1 [PhCH_2_CH(OMe)]^+^	50	200	39
			104.9 [PhCH_2_CH_2_]^+^	50	200	38
MC-YR	24.0	1045.2 [M+H]^+^	213.0 [Glu-Mdha+H]^+^	150	250	62
			135.1 [PhCH_2_CH(OMe)]^+^	150	250	63
MC-LR	25.1	995.5 [M+H]^+^	213.0 [Glu-Mdha+H]^+^	150	200	73
			135.1 [PhCH_2_CH(OMe)]^+^	150	200	62
MC-LW	34.9	1025.4 [M+H]^+^	446.1 [C_11_H_15_O-Glu-Mdha-Ala]^+^	150	200	30
			375.1 [C_11_H_15_O-Glu-Mdha]^+^	150	200	25
OA	37.0	827.6 [M+Na]^+^	809.4 [M+H-H_2_O]^+^	75	200	50
			723.6 [M+H-3H_2_O-CONa]^+^	75	200	50

* hypothesized formula for product ion from ref [24], except for okadaic acid (OA).

**Table 3:** Method Detection Limits (MDLs) and Method Quantification Limits (MQLs) for each compound in different matrices.

Toxin	Lake Water		River Water		Sea Water	
	MDL (ng L^−1^)	MQL (ng L^−1^)	MDL (ng L^−1^)	MQL (ng L^−1^)	MDL (ng L^−1^)	MQL (ng L^−1^)
DA	19	58	29	88	14	44
MC-RR	3	9	0.4	1	2	6
MC-YR	4	13	6	17	2	6
MC-LR	5	16	2.	6	0.3	1
MC-LW	4	11	17	50	6	20
OA	2	5	0.8	2	0.5	2

## Supplementary Materials

The following are available online at https://www.mdpi.com/1660-4601/17/13/4782/s1. Table S1: List of target MC variants, Table S2: Physical-chemical parameters of the water samples, Table S3: Mean recovery (%) in tap water (500 mL) at different concentration levels (400 and 100 ng L^−1^), Table S4: ME in the different matrices investigated for each analyte. Figure S1: Chemical Structure of DA and its isomers, Figure S2: A typical MRM chromatogram of a standard solution (200 μg L^−1^).
