# Supplementary material for: Simultaneous Pre-Concentration and HPLC-MS/MS Quantification of Phycotoxins and Cyanotoxins in Inland and Coastal Waters
Source: Int J Environ Res Public Health. 2020 Jul 3;17(13):4782. doi: 10.3390/ijerph17134782 (PMC7369962; doi:10.3390/ijerph17134782)
Supplement: Supplementary file 1 [file ijerph-17-04782-s001.pdf]

International Journal of
Environmental Research and Public Health

MDPI

Supplementary Material

Simultaneous Pre-concentration and HPLC-MS/MS Quantification of Phycotoxins and Cyanotoxins in Inland and Coastal Waters

Francesca Merlo [1], Federica Maraschi [1], Davide Piparo [1], Antonella Profumo [1,*] and Andrea Speltini [2]

[1] Department of Chemistry, University of Pavia, via Taramelli 12, 27100 Pavia and Italy; francesca.merlo02@universitadipavia.it (F.Me.); federica.maraschi@unipv.it (F.Ma.); davide.piparo01@universitadipavia.it (D.P.)

[2] Department of Drug Sciences, University of Pavia, via Taramelli 12, 27100 Pavia, Italy; andrea.speltini@unipv.it

* Correspondence: antonella.profumo@unipv.it

Table S1. List of target microcystin (MC) variants.

Microcystin	X	Z
MC-LR	Leucine	Arginine
MC-LW	Leucine	Tryptophan
MC-RR	Arginine	Arginine
MC-YR	Tyrosine	Arginine

Table S2. Physical-chemical parameters of the water samples.

	Tap water	Mergozzo Lake Water	Garda Lake Water	Staffora River	Ligurian Sea Water
pH	7.7	7.8	7.1	7.9	8.1
Conductivity at 20 °C ($\mu S\ cm^{-1}$)	270	59	278	293	5×10^4
Cl^- (mg L^{-1})	5.0	2.2	13.3	3.9	19000
NO_3^- (mg L^{-1})	0.6	3.0	9	1.6	< 0.05
SO_4^{2-} (mg L^{-1})	5.0	6.0	14.3	12.9	2650
Ca^{2+} (mg L^{-1})	36.1	7.0	39	54	400
Mg^{2+} (mg L^{-1})	8.1	1.6	7.2	7.8	1260
Na^+ (mg L^{-1})	12.5	2.7	9.5	5.4	10600

Table S3. Mean recoveries (%) in tap water (500 mL, EF=200) at different concentration levels (400 and 100 ng L^{-1}).

	Mean Recoveries (%)	
	**400 ng L^{-1}**	**100 ng L^{-1}**
DA	80	74
MC-RR	101	106
MC-YR	119	91
MC-LR	116	95

MC-LW	76	70
OA	95	120

RSD < 10%, n = 3.

Table S4. Matrix effect (ME) (%) in environmental matrices for each analyte.

	Lake Water	River Water	Sea Water
DA	203	116	452
MC-RR	88	77	180
MC-YR	108	65	418
MC-LR	113	63	212
MC-LW	114	61	476
OA	98	63	142

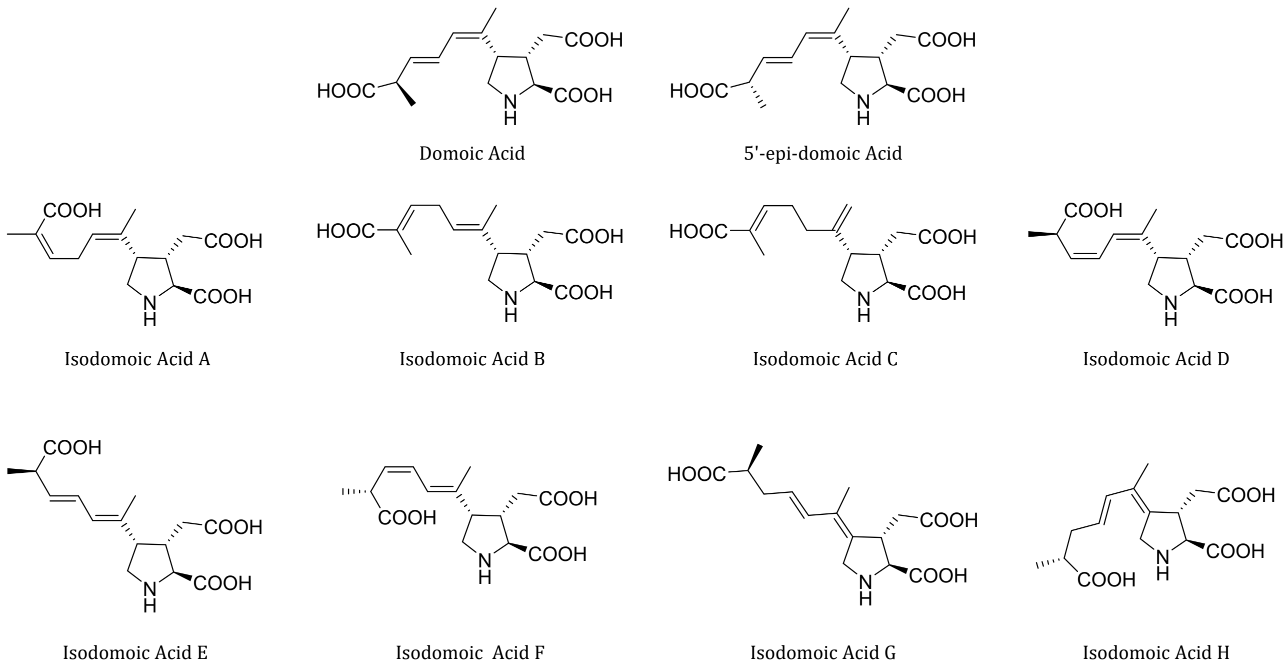

Figure S1. Chemical Structure of domoic acid (DA) and its isomers.

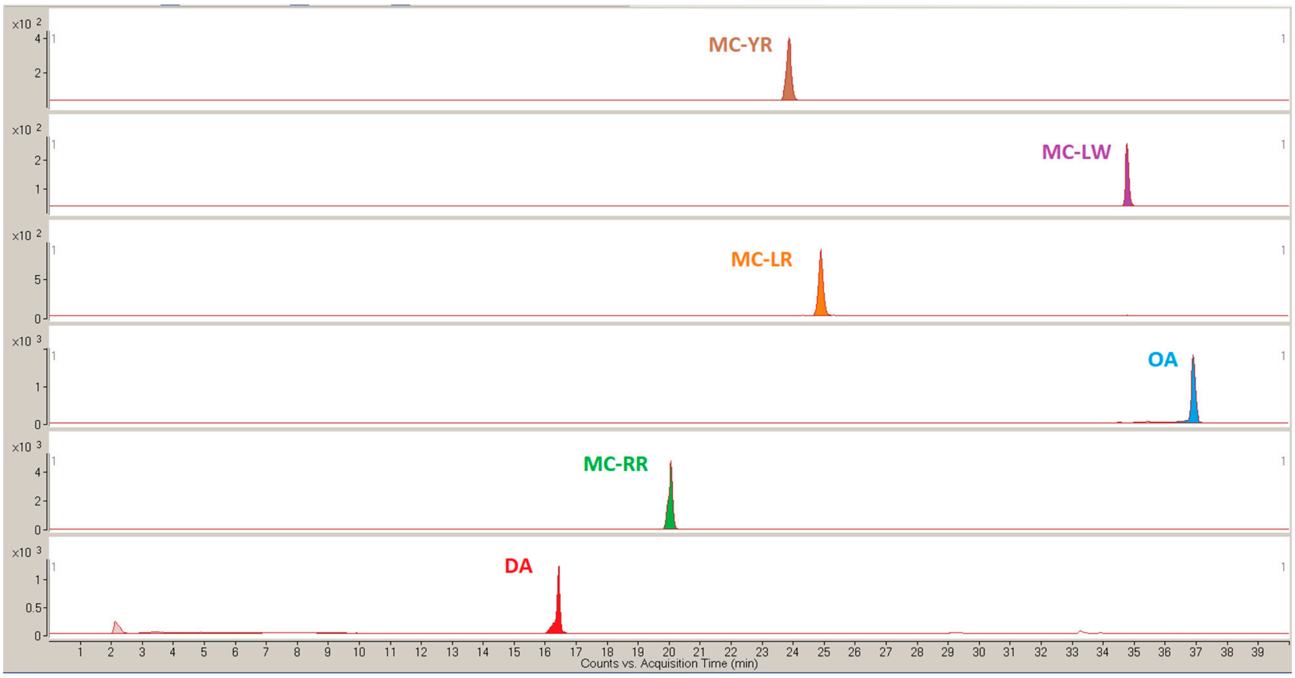

Figure S2. A typical Multiple Reaction Mode (MRM) chromatogram of a standard solution (200 μg L^{-1}).
